# Case Report: Revaluation of a previously diagnosed hazelnut allergy with component resolved diagnosis (CRD)

**DOI:** 10.1186/2045-7022-4-S2-P39

**Published:** 2014-03-17

**Authors:** Francesca Saretta, Roberto Perini, Elio Tonutti, Daniela Visentini

**Affiliations:** 1 Pediatric Department, Hospital of Palmanova, A.S.S. 5 "Bassa Friulana", Via Natisone 1, Palmanova, 33057, Italy; 2 Hospital of Palmanova, A.S.S. 5 "BAssa Friulana", Pediatric Department, Palmanova, Italy; 3 Azienda Ospedaliero-Universitaria di Udine Diagnostic Allergology and ImmunoPathology Departm, Udine, Italy

## Introduction

We report a case of an eleven years old girl who was referred to our Allergy Outpatient Clinic for the revaluation of a hazelnut allergy. During her infancy she was successfully desensitised to milk and egg. Afterward she had been followed by another Allergy Unit where, according to skin prick test and specific IgE dosage, it had been suggested to strictly avoid peanut and all treenuts, although clinical history was not suggestive of treenuts allergy. She was also provided with an auto-injectable adrenaline, due to the high risk of severe reactions to treenuts. No oral provocation test was performed to validate diagnosis and adrenaline prescription.

## Methods and Results

At her first visit in our Allergy Outpatient Clinic, the girl's mother showed us an ISAC (Immuno Solid-phase Allergen Chip) test, suggested by the colleague who performed the specific IgE dosage to better clarify the allergological results. On this test, a moderate positivity to dust mites, hazel, birch and grass pollen, a moderate positivity to *Jugr3* and only a mild positivity to *Cora1* were identified. All other peanuts and treenuts allergens were negative. A prick test for hazel pollen (positive, 5 mm) was performed; hazelnut extract , hazelnut with prick-by-prick method all resulted negative. Therefore an oral provocation test was performed with hazelnut and no immediate reactions were observed. In the following days she ate hazelnut without any symptoms.

## Conclusion

In this case-report we highlight the important role of CRD in the diagnosis of hazelnut allergy. Moreover, we underline the importance of a correct diagnosis of food allergy which has to rely not only on specific IgE dosage or skin prick test, but should be confirmed by an oral provocation test.

